# Just fun or a prejudice? – physician stereotypes in common jokes and their attribution to medical specialties by undergraduate medical students

**DOI:** 10.1186/s12909-017-0964-6

**Published:** 2017-07-26

**Authors:** Sigrid Harendza, Martin Pyra

**Affiliations:** 0000 0001 2180 3484 grid.13648.38 III. Medizinische Klinik, Universitätsklinikum Hamburg-Eppendorf, Martinistr. 52, D-20246 Hamburg, Germany

**Keywords:** Identity formation, Medical specialties, Prejudice, Professionalism, Stereotype, Undergraduate medical education

## Abstract

**Background:**

Many jokes exist about stereotypical attributes of physicians in various specialties, which could lead to prejudices against physicians from a specific specialty. It is unknown whether and when medical students are aware of stereotypes about different specialties. The goal of this study was to analyze the degree of stereotypes that exist about medical specialties amongst undergraduate medical students at different stages of their education.

**Methods:**

One hundred fifty-two jokes with different content about attributes of physicians from different specialties were found by an internet search. In total, 36 characteristics of the five specialties of anesthesia, general surgery, internal medicine, orthopedics, and psychiatry were extracted from the jokes and they constituted the basis for the development of an online questionnaire. The questionnaire allowed each characteristic to be assigned to one of the five specialties and was sent to 999 undergraduate medical students from semester 1, 7, and 12 at the Medical Faculty of Hamburg University.

**Results:**

Three hundred eight (30.8%) of the invited students completed the survey. The characteristics of general surgeons and psychiatrists were assigned congruently most frequently (>50%). For internists and orthopedics, there was a significantly more congruent assignment of the characteristics by final year students versus students in their first semester. Male students assigned the characteristics of anesthetists and internists significantly more congruently than female students. The three characteristics “…are a bit slow on the uptake”, “…consider income to be relatively unimportant”, and “...apologize a lot” were not assigned to any of the five specialties by more than 50% of the students.

**Conclusions:**

While stereotypes about physicians from certain specialties seem to exist commonly, medical educators need to be aware that stereotypes about specialties might develop during undergraduate medical training. In order to support students in their professional identity formation without developing stereotypes, medical educators should receive training. Performing a similar study with physicians in postgraduate training would shed some light on stereotypes and prejudices that might develop at a later stage in medical education.

## Background

Finding the right specialty for postgraduate medical training seems to be a difficult process for undergraduate medical students [1]. To provide orientation in the jungle of medical specialties some medical schools offer career-development courses or programs for undergraduate medical students even starting as early as in the preclinical years [2, 3]. Physician shadowing experiences have also been shown to increase medical students’ interest in a specialty [4]. Aside from serious recommendations, a funny algorithm was created in 2005 by a resident from Yale School of Medicine to guide students’ choice of specialty based on “personality characteristics” [5]. While it included “recommendations” for ten different medical specialties, this algorithm was posted in a blog of a professional networking website for doctors and medical students [6] in 2011 with its “recommendations” extended to 18 specialties. Anecdotal evidence reports residents and medical students to find some truth in the depicted personality characteristics with respect to their assigned specialties [5]. This raises some concerns, as not many of the personality characteristics are flattering and role modelling has been shown to play the greatest role in specialty choice [7, 8]. Furthermore, it remains unclear whether funny stereotyping nurtures common prejudices or describes some hidden unfavorable truths about physicians with certain characteristics favoring certain specialties.

Humor can be used to reduce nervousness, and anxiety and is frequently used in the medical workplace [9] and physicians use it in particular as a strategy to avoid burnout [10] or to manage uncertainty [11]. Gallows humor, i.e. humor under serious circumstances, has been shown to help physicians to reduce stress in situations they cannot change, e.g. a patient’s death [12]. During medical education, humor applied by teachers can help students to relax, for instance, if they are not able to answer a question they have been asked [13]. Derogatory humor or stereotyping can be encountered in the medical environment with respect to many different groups of people: between doctors and nurses [14], between clinicians and scientists [15], against patients [16], against women [17], against homosexuals [18], and against different medical specialties [5, 6, 19–21]. Medical students become more cynical as they move through their undergraduate training [22]. Additionally, cavalier humor beliefs, i.e. a lighthearted, less serious, uncritical approach toward humor that dismisses potential harm to others, has been demonstrated to facilitate the expression of group dominance [23]. This might in fact worsen the humorous effect, which is potentially offensive to someone by its nature anyway. Many jokes have been described recently wherein doctors are caricatured in an unflattering way to enhance the humorous effect [24, 25]. Such jokes might contribute to the declining status and authority of physicians in general [26] and to influencing career choices of medical students [27]. Gutmann and Salzmann even hypothesize that jokes about different specialties – when viewed as being innocuous and shared with medical students – might even promulgate negative stereotypes to the extent of manipulating medical students’ interaction with and perception of physicians from various fields [28].

In 2004, a qualitative study extracted professional stereotypes associated with general medicine and surgery from Brazilian medical residents [29] while already in 1999, concerns were raised that negative specialty stereotypes could persuade medical students not to choose those specialties for postgraduate training [28]. This concern is cued by the mere recognition that a negative group stereotype could apply to oneself in a given situation [30]. This phenomenon is called “stereotype threat” and has been demonstrated, for example, with a study on underachievement of UK medical students from ethnic minorities [31]. Furthermore, medical students have been reported to be deterred from a surgical career due to negative stereotypes of surgeons [32] and some specialties e.g. rheumatologists, predict a shortfall of residents in their specialty in the coming years [33]. Hence, it might be worthwhile to take a closer look at how medical specialties are stereotyped in common jokes and whether medical students recognize these stereotypes. It is unknown, whether stereotypes characterizing physicians from different specialties exist in general or might develop during undergraduate medical education.

With this study, we were interested in answering the following questions: 1) Which characteristics assigned to medical specialists in common doctor jokes are assigned by medical students from different years of undergraduate training to the respective specialty? 2) Is there a difference between the years of training with respect to the assignment of characteristics to a certain specialty? 3) Does a difference between male and female students exist with respect to the assignment?

## Methods

### Internet search for doctor jokes including at least one medical specialty

To identify doctor jokes involving medical specialties we searched the internet using the Google search engine between August and October 2013. The search was performed separately in English and in German, which included the following search terms: “doctor jokes” or “jokes” in combination with “the respective name of any medical specialty available at Hamburg University Medical Center”. Only publicly available sites were included. When an identified site included a link to another site with doctor jokes, this link was searched as well. Only jokes including at least one medical specialty were copied from the sites for further analysis. The search was discontinued when no new jokes were found with this search strategy.

The internet yielded 167 doctor jokes including at least one medical specialty, resulting in 152 jokes (83 in German, 69 in English) after the elimination of jokes with similar content. The 152 jokes were then further analysed. The following specialties occurred in the jokes: general surgeons (57×), psychiatrists (33×), gynaecologists (23×), orthopaedics (18×), anesthetists (16×), internists (16×), pathologists (8×), radiologists (8×), heart surgeons (6×), neurologists (6×), plastic surgeons (6×), ophthalmologists (5×), dermatologists (5×), general practitioners (4×), urologists (4×), ENT (3×), cardiologists (3×), proctologists (3×), and neurosurgeons (2×). For further analysis, we included only specialties with more than 10 mentions. Jokes including gynaecologists were excluded from further analysis despite their high number of mentions because jokes included mostly sexist punchlines involving female body parts or sexual acts.

### Definition of specialty characteristics from the jokes

From the jokes including anesthetists, general surgeons, internists, orthopaedic surgeons, and psychiatrists both authors individually extracted personality characteristics, which were assigned to a respective specialty by a respective joke. Personality characteristics were then compared, discussed, clarified, and combined when the very similar characteristic for a certain specialty occurred in several jokes. Individual characteristics were only included when complete agreement was reached between the authors. A total number of 36 characteristics could be identified and assigned to the respective specialties: anesthetists (6), general surgeons (7), internists (8), orthopaedic surgeons (8), and psychiatrists (7) (Table 1).

**Table 1:** Stereotypes extracted from the jokes

Characteristics per specialty	Number of jokes per characteristic
Anesthetists	
are always on a break.	5
often appear to be idle.	5
are a bit slow on the uptake.	4
only look at patients with “algorithms”.	3
keep patients alive.	2
administer treatments that are wearing on the patient.	1
General Surgeons	
are interested in a high income.	13
act first, think later.	12
are narcistic.	11
are authoritative and boss others around.	9
are often decisive.	6
strongly think hierarchically.	5
rarely look into the patient’s chart.	1
Internists	
rarely cure.	9
regard themselves as being particularly intelligent.	8
are the keenest thinkers of all doctors.	6
ponder everything.	6
are indecisive.	3
are hesitant and have a “wait and see attitude”.	3
have a lot of knowledge but few skills.	2
consider income to be relatively unimportant.	1
Orthopedics	
know little beyond their specific field of expertise.	17
rarley use their brain.	16
rarely refer to books.	4
work hard physically.	2
usually require more time for a task than they say they will need.	2
hardly ever take diagnoses made by others into account.	2
hardly ever use lab results.	1
are good looking and physically fit.	1
Psychiatrists	
take statements from others very seriously.	11
vare empathetic and understanding.	7
sometimes have the same problems as their patients.	5
sometimes misjudge the success of their treatment.	5
can only cure when a patient cooperates.	2
apologize a lot.	1
paraphrase without answering.	1

### Development of an online questionnaire

The 36 characteristics were used in a randomized order without the specialty mentioned at the beginning of the individual sentences. Every statement was followed by six boxes labelled “anesthetists”, “general surgeons”, “internists”, “orthopaedics”, “psychiatrists”, and “none”. Participants were asked whether they felt a statement could be assigned to one of the respective specialties or not and to tick the box of their choice for every statement (Original text from the questionnaire: Below you will find stereotypic statements about physicians. Please mark one box for the following statements, e.g.: … act first, think later. Anesthetists, general surgeons, internists, orthopaedics psychiatrists, none of these specialties). Only one box per statement could be marked. The questionnaire could only be completed when all items were answered.

### Participants

In May 2014, all undergraduate medical students from semester 1 (S1, *n* = 391), semester 7 (S7, *n* = 402), and semester 12 (S12, *n* = 206) received a link to the questionnaire by email. At the time of the study, our medical school had a traditional curriculum with clinical courses starting in semester 5 and a final practice year starting in semester 11. Students were informed that the goal of this study was to identify their opinion about characteristics of physicians from different medical specialties. Participation was anonymous and voluntary. With their replies, students gave informed consent for participation and disclosed their sex and semester of undergraduate training. The study was performed in accordance with the Declaration of Helsinki and the Ethics Committee of the Hamburg Chamber of Physicians had waived ethics approval for this study. Of the 999 distributed questionnaires, 308 completed questionnaires (*n* = 116, male; *n* = 192, female) were returned resembling a return rate of 30.8% (S1: *n* = 124; *n* = 47, male; *n* = 77, female; return rate 31.7%; S7: *n* = 123; *n* = 50, male; *n* = 73, female; return rate 30.6%; S12: *n* = 61; *n* = 19, male; *n* = 42, female; return rate 29.6%).

### Analysis

Statistical analyses were performed using IBM SPSS Statistics 22.0 (SPSS Inc., Chicago, USA). For the analyses, only participants with complete data sets were included and the answers were transformed from numbers to percentages per year of study. We performed a univariate ANOVA with the independent variables of students’ sex, semester of study, and physician specialty while the dependent variable was the congruent assignment of a characteristic to the specialty it was extracted from originally. The level of significance was set to *p* < 0.05.

## Results

**Fig. 1:**
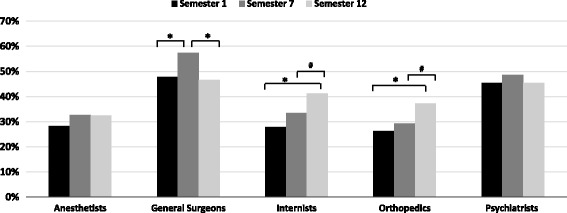
Percentage of characteristics assigned to a specialty by semester; *: *p* ≤ 0.001, ^#^: *p* < 0.05

General surgeons and psychiatrists were the specialties with the most congruent assignment of characteristics (Fig. 1). A significantly greater congruent assignment of characteristics was observed for internists in S12 versus S1 (*p* < 0.001) and in S12 versus S7 (*p* < 0.05) as well as for orthopaedics in S12 versus S1 (*p* < 0.001) and in S12 versus S7 (*p* < 0.05). For general surgeons, students from S7 assigned the characteristics most congruently versus S1 (*p* < 0.05) and S12 (*p* < 0.05). No significant differences were observed for characteristics of anesthetists and psychiatrists between the congruent assignments by students from the different semesters.

**Fig. 2:**
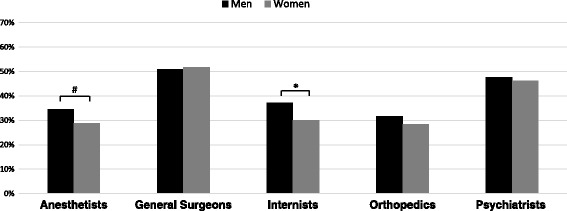
Percentage of characteristics assigned to a specialty by male and female students; *: *p* = 0.001, ^#^: *p* = 0.011

**Fig. 3:**
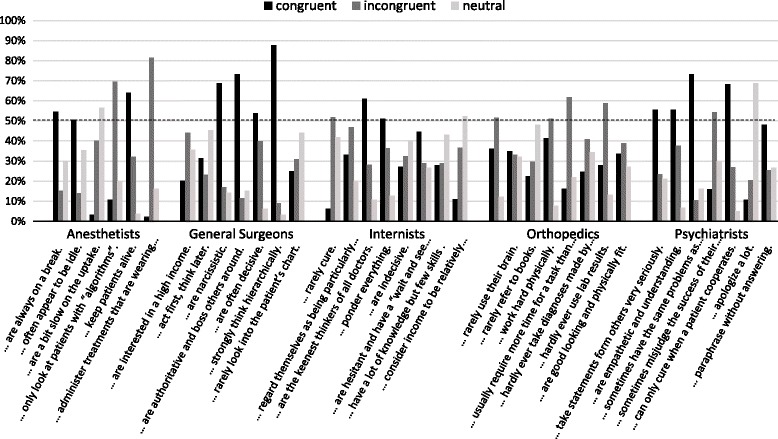
Percentage of congruent, incongruent, and neutral assignment per characteristic

Male students assigned the characteristics of anesthetists (*p* = 0.011) and internists (*p* = 0.001) significantly more congruently than female students (Fig. 2) while no significant differences were observed for the assignment of characteristics to general surgeons, orthopaedics, and psychiatrists between male and female students. Figure 3 shows the congruent, incongruent or neutral assignments for the different characteristics to the specialties by all students. The characteristics “…strongly think hierarchically.” (general surgeons) and “…sometimes have the same problems as their patients.” (psychiatrists) were assigned with the highest rates of congruency. The characteristics “…keep patients alive.” (anesthetists) and “… are the keenest thinkers of all doctors.” (internists) received congruent assignment rates above 50% in their respective specialty. For orthopedics, no characteristic was assigned above 50% congruently. Three characteristics received a neutral assignment above 50%: “… apologize a lot.” (psychiatrists), “… are a bit slow on the uptake.” (anesthetists), and “…consider income to be relatively unimportant” (internists).

## Discussion

The undergraduate medical students assigned more than half of the stereotypic characteristics extracted from the jokes congruently to the specialty of their origin. There was no increase in assignment of the stereotypic characteristics to surgeons and psychiatrist by students from different semesters, which could suggest that these stereotypes might be common knowledge, e.g. from movies or books and widely spread [29, 34]. For internists and orthopedics, the number of congruently assigned characteristics increased significantly with the number of semesters at medical school, suggesting that some of the stereotypes might develope during undergraduate training.

Congruent assignment of internists’ characteristics increased significantly from S1 to S12 with the most congruent assignment of the characteristic “… are the keenest thinkers of all doctors” by 31.1% in S12. During their undergraduate studies, medical students become increasingly exposed to clinical reasoning processes and especially observe residents in their daily practice of how they reason and what they reason about [35]. This might also explain why “… ponder everything.” and “… are hesitant and have a ‘wait and see attitude’.” reached high assignment scores as well. General internists were characterized by internal and surgical residents as having intellectual skills, being meticulous and attentive to details, being slow to resolve problems and making decisions, and working with probabilities and hypotheses [29], which underscores character traits extracted from the jokes. Male students assigned characteristics accredited to internists significantly more congruently to internists than female students who assigned the characteristics that include intelligence and brainpower much more incongruently to general surgeons. Surgery has been regarded to be a masculine specialty [32] and women tend to regard intelligence as a male attribute [36]. This might be one of the underlying reasons why female students assigned characteristics involving intelligence less congruently to internists.

A significant increase in congruent assignments between S1 and S12 was observed for characteristics of orthopedics, suggesting that stereotypes of orthopedics are not common in the public but increase during undergraduate medical training when students have more contact with orthopedics and other disciplines. The so-called “in-group bias”, which describes how stereotypes and their affective responses, i.e. prejudices, develop, when one feels social affiliation with one group and attributes certain characteristics to another group that one does not wish to belong to [37], might play a role for the increase in congruency, if, for instance, more advanced medical students favor other specialties than orthopedics because of several of the negative stereotypes associated with this specialty. Orthopedics’ characteristics from the jokes showed the highest rate of incongruent assignments to general surgeons and a very low rate of incongruent assignments to internists. This might be due to a stereotypic distinction by the students between operating and non-operating specialties. In a self-assessment study of surgeons and physicians, surgeons assessed themselves as significantly more extroverted, less neurotic, and more intolerant of uncertainty than physicians [38].

Only half of the characteristics of anesthetists were assigned congruently by more than 50% of the students. Hence, it can be assumed, that stereotypes about anesthetists are not widely spread in the public. The anesthetist is only visible to a patient for a short period of time [39], which might be too short to develop stereotypes. When asked about the role of an anesthetist, 45% of patients from a Swiss hospital who underwent an elective operation thought that anesthetists worked under the supervision of the surgical team [39]. This was also one aspect that was found in the jokes. No increase in congruent assignments was observed between S1 and S12, which might be because undergraduate medical students seem to have very little teaching contact with anesthetists [40] unless they choose anesthesiology as an elective. It can be seen from the jokes, that many punchlines on anesthetist characteristics stem from the interaction of surgeons and anesthetists, which is a lot closer during residency and therefore postgraduate education seems to be more prone for the development of stereotypes. Female students assigned characteristics of anesthetists even significantly less congruently than male students in our study. This might be related to certain gender stereotypes among medical students towards anesthesiology, which lead to an underrepresentation of women in emergency medicine [41].

The characteristics that have been assigned to the category “neutral” by the majority of the students included mostly negatively connoted statements like “… rarely look into the patient’s chart.” or “… rarely refer to books.”. These stereotypes are in contrast to medical students’ image of the ideal physician they would like to become who comprises characteristics like reliability, trustworthiness, thoroughness, and congeniality [42]. Students in our study might have marked the negative characteristics with “neutral” because they might wish to choose one of the five specialties for postgraduate training and wish to stick to their own image of an ideal physician, even though students in another study describe some physicians they met during their undergraduate training as negatively deviating from this ideal [42]. However, during postgraduate education trainees begin to identify with the specialty of their respective training and might develop a stronger awareness of characteristics of other specialties in close interaction, e.g. in surgery and anesthesiology. This might result in creating jokes about the other specialty, leading to cavalier humor, which dismisses its potential harm to the less dominant group who is the target of the pun [23]. In the group-dominance model of humor, social dominance motives predict favorable reactions towards jokes targeting other groups [23]. The “superiority of humor” theory describes that jokes are experienced to be funnier, if they portray the group one identifies with as “victorious” [43]. In these respects, jokes about medical specialties can be potentially harmful by distributing stereotypes and creating prejudices, which might even lead to medical students not choosing a particular specialty for postgraduate training.

Our study has several limitations. The collection of jokes might be incomplete due to our search strategy and the phrasing of characteristics we extracted from the jokes was not validated for the questionnaire. Therefore, we cannot exclude that a study participant might have marked a characteristic as “neutral” because the phrasing was not comprehensible. Furthermore, the number of characteristics per specialty was either six, seven or eight. This might have led to a stronger influence of the individual characteristic on the overall result for the specialties with the smaller number of characteristics, because a similar number of congruent answers led to a different percentage of congruent answers per specialty (e.g. two out of six, i.e. 30%, versus two out of eight, i.e. 25%). In this pilot study, only students from one medical faculty participated which might have led to a bias caused by the particular culture at this faculty and the number of students per semester differed with the smallest number in S12. Even though the return rate was about 30% in all semesters, the absolute number of students who were compared in the three groups was not similar. Like in our study, unfortunately medics tend to show lower response rates in surveys compared with other populations [44] and a decline in return rates of social sciences surveys has been described in general [45]. Furthermore, the generalizability of our study is hampered by the fact that age distribution of medical students might be different in other countries with respect to the semester in medical school and age was not included in the sociodemographic aspects of the questionnaire. Additionally, an overlap of characteristics could be noticed for the surgical disciplines general surgery and orthopedics, which might be the cause for incongruent assignments to these specialties. However, even though the percentage of congruent assignments of characteristics to orthopedics was still below 40% in S12, the significant increase compared with S1 might still be due to exposure to orthopedics during undergraduate medical training. Even though it appears from our data that some stereotypes, e.g. towards surgeons or psychiatrists, seem to be common knowledge, the increase in stereotyping of internists and orthopedics with a growing number of semesters suggests that some stereotypes develop during undergraduate training. Since our study is a cross-sectional study, it is another weakness that career choices of our participants will remain unknown.

As it has been shown that specialty stereotypes can influence medical students’ choice of career [28, 32], medical educators need to be aware that their students regard them as role models for their respective specialty [46]. Improving the perception of a specialty might also be a useful action against possible prejudices based on stereotypes [47]. Furthermore, professional behavior can be taught and even has an influence on patient outcome as has been demonstrated in a study with anesthesiology residents [48]. In professional medical conduct, humor has its place to reduce stress and enhance teamwork, yet it needs to be used in a non-humiliating manner [49]. Additionally, medical educators need to be trained to support students in their professional socialization and identity formation [50]. If medical students learn about the negative effects the dissemination of stereotypes about specialties can have on identity formation [28], they might learn to handle prejudices and to use humor in a professional way.

## Conclusions

In conclusion, our study shows that some stereotypes about general surgeons and psychiatrists are present in medical students from different semesters, reflecting common beliefs, while stereotypes about internists and orthopedics seem to emerge with the time spent at medical school. As jokes about the latter specialties reflect beliefs, which seem to be partially socialized during undergraduate medical training and may distort student career predictions, medical educators need to be aware of this problem and should support students in their professional identity formation without developing stereotypes. A similar survey with the characteristics of specialties tested in our study should be conducted with physicians to become aware of the occurrence of stereotypes in postgraduate training and to be able to define supportive measures for better etiquette in teams. Additionally, focus groups with students should be conducted to explore the identified stereotypes with respect to students’ perceptions and a possible influence on their career choices.
